# Definitive carbon ion radiotherapy for thoracic bone and soft tissue sarcoma

**DOI:** 10.1186/s12885-026-16541-x

**Published:** 2026-07-15

**Authors:** Jian Chen, Ningyi Ma, Yaqi Li, Kai-Liang Wu, Jingfang Mao

**Affiliations:** Department of Radiation Oncology, Shanghai Proton and Heavy Ion Center, Fudan University Cancer Hospital, Shanghai Key Laboratory of radiation oncology (20dz2261000), Shanghai Engineering Research Center of Proton and Heavy Ion Radiation Therapy, Shanghai, 201321 China

**Keywords:** Thoracic bone and soft tissue sarcoma, Sarcoma, Radiotherapy, Carbon ion radiotherapy

## Abstract

**Background:**

Thoracic bone and soft tissue sarcoma (BSTS) represents a rare malignancy. For patients with medically inoperable BSTS, radiotherapy (RT) is a recommended treatment modality. Carbon ion radiotherapy (CIRT) has shown superior efficacy compared to photon-based RT. However, data on the use of CIRT for thoracic BSTS remain scarce. This study aimed to evaluate the clinical outcomes and toxicity profile of CIRT in patients with thoracic BSTS.

**Methods:**

The inclusion criteria were as follows: 1) ≥ 14 years old; 2) primary or metastatic BSTS in chest area, with histologically confirmed primary tumor; 3) with visible lesion; 4) definitive CIRT intent; and 5) had at least one follow-up, and the time between follow-up and the first fraction of CIRT was longer than 1 month. The prescribed CIRT doses ranged from 59.5 to 80 Gy (RBE) delivered in 10–23 fractions. Treatment-related toxicities were assessed using the Common Terminology Criteria for Adverse Events Version 4.03, while tumor response was evaluated according to the Response Evaluation Criteria in Solid Tumors Version 1.1. Local control (LC), progression-free survival (PFS), and overall survival (OS) rates were estimated using the Kaplan-Meier method. Subgroup analyses were performed using the Log-rank test.

**Results:**

A total of 33 patients with 35 lesions were included in the study. The median age of the cohort was 56 years (range: 22–86 years), with a median follow-up duration of 42.1 months (range: 3.8–100.6 months). The 3-year LC rates for the entire cohort were 85.2%. There was no statistically significant difference in LC when categorized by median tumor volume and biological effective dose. The use of simultaneous integrated boost technology appears to improve the LC. Among patients with primary thoracic BSTS, the 3-year LC, PFS， and OS rates were 84%, 54.8%， and 76.7%, respectively. Patients with postoperative recurrence (PORC) demonstrated significantly inferior PFS and OS. Except one case of grade 3 chest wall pain and 1 brachial plexopathy, no grade ≥ 3 toxicities were observed.

**Conclusions:**

CIRT is safe and effective in the management of thoracic BSTS. Further studies with more cases and longer observation periods are warranted.

**Trial registration:**

Retrospectively registered on 13th February, 2026; ChiCTR2600118943; Chinese Clinical Trial Registry; https://www.chictr.org.cn/bin/project/edit?pid=308155.

## Background

Bone and soft tissue sarcomas (BSTS), which constitute approximately 1% of all adult malignancies, represent a rare and heterogenous disease group. Thoracic BSTS can originate from diverse structures such as the chest wall, mediastinum, heart, spine, or pleura. While surgery remains the primary treatment [1], definitive radiotherapy (RT) is the main alternative for patients who are not surgical candidates. Unfortunately, the efficacy of photon radiotherapy (XRT) is often unsatisfactory, as 3-year local failure could exceed 40% even with modern RT techniques [2].

Carbon ion radiotherapy (CIRT) offers distinct physical and biological advantages for treating BSTS. Physically, its Bragg peak and narrow penumbra enable precise dose delivery with superior normal tissue sparing. Biologically, carbon ions yield a high relative biological effectiveness (RBE), enhancing radioresistant cell kill. This synergy makes CIRT a promising modality for improving tumor control while minimizing toxicity to surrounding organs at risk (OARs) [3].

CIRT has demonstrated superiority to photon therapy for BSTS [4, 5]. For example, in patients with retroperitoneal soft tissue sarcomas treated with CIRT, the rates of 5-year local control (LC) and overall survival (OS) were 69% and 50%, respectively. No gastrointestinal complications were observed, and no treatment-related toxicities of grade 2 or higher were reported. However, existing clinical data were largely derived from extrathoracic sites. The unique anatomical and dosimetric challenges of thoracic BSTS (e.g., originating from or metastasizing to thoracic organs or mediastinal structures), with its specific OARs and corresponding dose constraints, mean that the efficacy and toxicity profile observed elsewhere cannot be directly extrapolated to thoracic lesions. Given the extreme rarity of thoracic BSTS and the lack of standard definitive treatments for both unresectable primary tumors and thoracic-limited metastases, CIRT is employed as a local definitive therapy with the shared goal of durable LC. To achieve adequate statistical power for evaluating the safety and efficacy of CIRT in this small patient population, we performed a combined analysis of these two groups, justified by their similar treatment intent and modality. Accordingly, we conducted a comprehensive retrospective analysis of 33 consecutive thoracic BSTS cases treated at our institution.

## Methods

### Patients and pretreatment evaluations

**Fig. 1:**
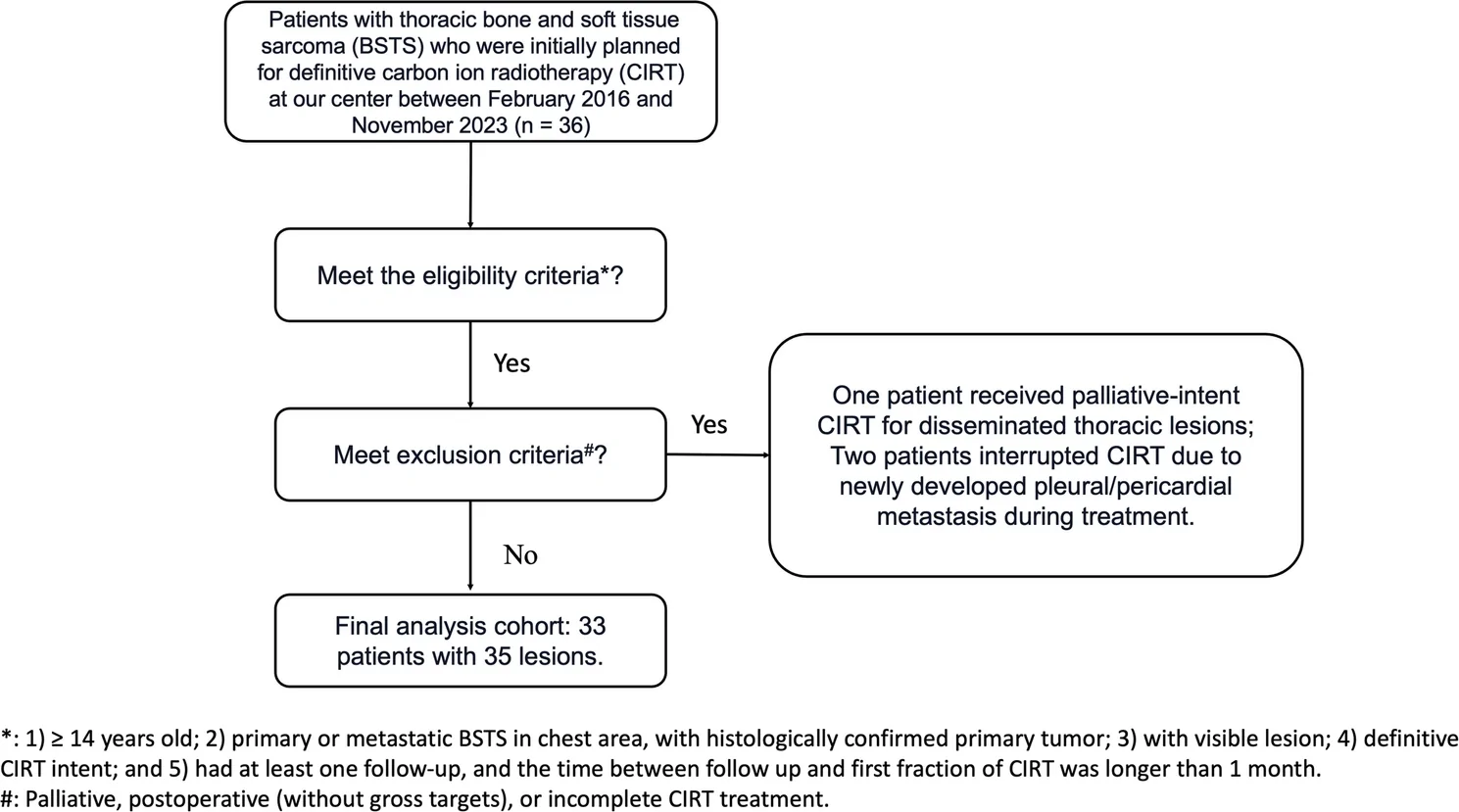
Flow diagram of patient screening, enrollment, exclusion and final analysis. Total initially planned patients: 36; Exclusions: 2 patients discontinued CIRT during treatment, 1 patient received palliative carbon ion radiotherapy; Final analytic cohort: 33 patients with 35 lesions

Patients who met the following criteria were enrolled in this study: 1) ≥ 14 years old; 2) primary or metastatic BSTS in chest area, with histologically confirmed primary tumor; 3) with visible lesion; 4) definitive CIRT intent; and 5) had at least one follow-up, and the time between follow-up and the first fraction of CIRT was longer than 1 month. Patients were excluded for palliative, postoperative (without gross targets), or incomplete CIRT treatment. A flow diagram summarizing patient screening, exclusion and final analytic population was provided as Fig. 1, in accordance with Strengthening the Reporting of Observational Studies in Epidemiology (STROBE) statement for observational retrospective studies. The study was approved by the Institutional Review Board (IRB) (approval number 260212EXP-02) of Shanghai Proton and Heavy Ion Center (SPHIC). Informed consent was obtained from all the participants.

Pretreatment evaluation in all patients included complete disease history and physical examination, complete blood count, electrocardiogram, pulmonary function tests, serum electrolytes, renal and liver function tests, and brain magnetic resonance imaging (MRI) or computer tomography (CT). A fluorodeoxyglucose positron emission tomography/computed tomography (FDG-PET/CT) scan for clinical staging was recommended, as we reported before in lung cancer and tracheobronchial adenoid cystic carcinoma research [6, 7].

### N/A

### Preparing, planning and delivery of CIRT

The procedures of CIRT, including preparing, planning and delivery, were performed similar to those previously described [6, 7]. The patients were immobilized in supine or prone position according to the location of tumour. The scanning scope of the simulation CT starts from the angle of the mandible to the adrenal glands to include tumour lesions, entire lungs, whole neck, and all the organs/tissues through which the beams were likely to pass. Tumour motion was evaluated using 4-dimensional (4-D) simulation CT. If tumour motion was < 5 mm, CIRT was performed under free breathing (FB); if the motion exceeded 5 mm, a breath control technique, either active breathing control (ABC, Elekta Oncology Systems, Crawley, UK) or respiratory gating (Anzai Respiratory Gating System, AZ-733 V, Anzai Medical Co. Ltd, Japan), was required to ensure the residual motion (RM) to < 5 mm during treatment.

The gross tumour volume (GTV) was determined according to contrast thoracic CT/MRI and PET/CT. For patients using the gating technique, an internal gross tumour volume (iGTV) was created by combining the GTVs of all respiratory phases. GTV/iGTV was generally expanded 1.5–4.0 cm longitudinally and 1.5 cm radially to form clinical target volume (CTV). This relatively large longitudinal margin was chosen based on the infiltrative, extensive, and multifocal growth pattern characteristic of BSTS. The exact margin within this range was individualized based on adjacent anatomical structures. Where natural barriers (e.g., pleura, fascia, vessels) could limit tumor extension, a smaller margin (toward 1.5 cm) was used; in regions lacking such barriers or along tissue planes that facilitate spread, a larger margin (up to 4.0 cm) was applied. This anatomy‑guided strategy balances coverage of microscopic spread with sparing of OARs, consistent with established sarcoma radiotherapy practice [8, 9]. The creation of planning target volume (PTV) included range uncertainties and set-up errors. In most instances, it was CTV plus a 0.3–0.5 cm lateral margin and a 0.5–1.2 cm margin along the beam direction.

All treatment plans were created using the Siemens Syngo treatment planning system with the built-in biological local effect model (LEM)-I, with the prescription dose expressed in Gy (RBE) as an RBE-weighted dose. The prescription doses were 59.5–80.0 Gy (RBE) per 16–23 fractions for non-hypofractionation and 60.0–65.0 Gy (RBE) per 10 fractions for hypofractionation, 5 fractions a week. Simultaneous integrated boost (SIB) technique could be used to escalate the dose to the center of the GTV in bulky tumors sparing surrounding OARs from excessive radiation exposure. For patients treated using the SIB technique, the dose administered to the SIB region was used for dose-response analysis in this study. When analysing the impact of dose on clinical outcomes, biological effective dose (BED) calculated by L-Q model was used.

The target coverage requirements were as follows: (i) at least 99% of the GTV was covered by 99% of the prescription dose (adherence to this requirement may be moderated to comply with the dose constraints for normal tissues), (ii) 99% of the CTV by 95% of the prescription dose, and (iii) 90% of the PTV by 90% of the prescription dose. The dose constraints to OARs for patients irradiated 16 fractions or more were as followed: (1) lung: mean dose (Dmean) of bilateral lungs < 14 Gy (RBE), the percentage volume of the lung receiving 20 Gy (RBE) or more in total lung (V20) < 20%, V5 < 50%; (2) maximum dose (Dmax) of the proximity bronchial tree (PBT) < 105% of the prescription dose or less than 80 Gy (RBE); (3) heart: V30 < 30%, V40 < 25%; (4) oesophagus: Dmean < 34 Gy (RBE), Dmax < 105% of the prescription dose or less than 80 Gy (RBE); (5) spinal cord: Dmax < 45 Gy (RBE); (6) brachial plexus Dmax < 60 Gy (RBE); and (7) stomach: Dmax < 50 Gy (RBE). The dose constraints to OARs for patients irradiated 10 fractions were as followed: (1) lung: Dmean of bilateral lungs < 5 Gy (RBE), V20 < 12%, V5 < 28%; (2) Dmax of PBT/esophagus/heart < 40 Gy (RBE); (3) Dmax of spinal cord/stomach < 30 Gy (RBE); (4) brachial plexus Dmax < 32 Gy (RBE).

### Follow-up and evaluation

This part was similar to previously described [6, 7]. All patients were evaluated weekly for treatment-related toxicities and disease response during treatment. After the completion of CIRT, all patients were required to be evaluated 3 months after the 1st day of CIRT, every 3–4 months within the first 2 years, every 6 months between years 3 and 5, and annually thereafter. Treatment-related toxicities were scored according to the Common Terminology Criteria for Adverse Events, version 4.03. Toxicities occurring 90 or more days after the initiation of CIRT were defined as late toxicities. Tumour response evaluation was evaluated using the Response Evaluation Criteria in Solid Tumors, version 1.1.

### Statistical analyses

The time to events was calculated from the start of CIRT until the first documented treatment failure. The LC, PFS, and OS were calculated using the Kaplan-Meier method. To avoid unit-of-analysis bias, lesion-based analysis was used for LC in the overall cohort and in patients with primary BSTS, while patient-based analysis was used for PFS and OS in patients with primary BSTS. The Log-rank tests were used to compare clinical outcomes between subgroups. Multivariate analysis was precluded due to limited sample size (*n* = 33). The Student’s *t*-test was used to compare differences between groups. All analyses were performed using SPSS^®^ statistics version 26 (Armonk, NY, USA).

## Results

### Patient characteristics

From February 2016 to November 2023, a total of 36 patients were initially planned for definitive CIRT. Among them, 3 patients were excluded before final outcome analysis: 2 patients interrupted CIRT due to newly developed pleural/pericardial metastasis during treatment, and 1 patient received palliative-intent irradiation for disseminated thoracic lesions. The remaining 33 patients with 35 lesions constituted the final analysis cohort (Fig. 1). The median age was 56 (22–86) years. The median maximum diameter of the lesions was 7.5 (range: 1.0–14.0) cm. Twenty-four patients had primary thoracic BSTS with 24 lesions. Among them, the most common histological types were liposarcoma (4 cases), synovial sarcoma (4), and aggressive fibromatosis (4). The predominant primary sites were the mediastinum (9 cases), chest wall (9), and lung (3). Of these 24 patients, 3 had a history of chest RT, and 14 had undergone prior thoracic surgery [with 11 cases representing postoperative recurrence (PORC) and 3 cases showing radiographically evident residual disease]. Nine patients had metastatic thoracic tumors, with leiomyosarcoma (3 lesions) being the most frequent histology and the lungs (10 lesions) representing the most common metastatic site. Three metastatic lesions of two patients had a history of chest RT. The patient characteristics are detailed in Table 1.

**Table 1:** Characteristics of patients and treatment

Characteristic	Entire cohort	Thoracic primary BSTS
No. of patients	33	24
Age at treatment, y		
Median	56.0	56.5
Range	22–86	22–86
Sex		
Male	24	18
Female	9	6
ECOG score		
0	27	19
1	6	5
Smoking history		
Yes	10	8
No	23	16
Chest RT history		
Yes	5	3
No	28	21
Maximum diameter of tumor, mm		
Median	75.0	82.6
Range	10.0–140.0	39.0–140.0
GTV Volume, cm^3^		
Median	111.41	130.0
Range	0.8–790.6	42.4–790.6
Stage*		
IA, T1N0M0 Gx	1	1
IB, T2N0M0 Gx	4	4
IB, T3N0M0 Gx	4	4
II, T1N0M0 G3	1	1
IIIA, T2N0M0 G2	1	1
IV, T2N0M1 Gx	1	1
T0N1M0 Gx	1	1
T1N1M0 Gx	2	2
T2N0M0 Gx	1	1
T2N0M0 G3	1	1
T3N0M0 Gx	4	4
T3N0M0 G3	1	1
T4bN1M0 Gx	1	1
Cannot be staged#	1	1
IV, T0N0M1 Gx	8	
IV, T0N0M1 G3	1	
Pathology type		
liposarcoma	5	4
synovial sarcoma	5	4
aggressive fibromatosis	4	4
chondrosarcoma	3	2
pleomorphic sarcoma	2	2
malignant peripheral nerve sheath tumor	2	2
fibrosarcoma	2	1
perivascular epithelioid cell sarcoma	1	1
Ewing sarcoma	1	1
pulmonary artery intimal sarcoma	1	1
malignant fibrous histiocytoma	1	1
hemangiosarcoma	1	1
leiomyosarcoma	3	0
alveolar soft part sarcoma	1	0
myofibroblastoma	1	0
Tumor location (lesions)		
Lung	13	3
Mediastinum	10	9
Chest wall	9	9
Esophagus	1	1
Heart	1	1
Sternum	1	1
Chemotherapy		
Yes	12	11
No	21	13
CIRT dose		
59.5–80 Gy (RBE) / 16–23 fractions	33 (lesions)	24
60–65 Gy (RBE) / 10 fractions	2 (lesions)	0

*BSTS: *thoracic bone and soft tissue sarcoma, *ECOG:* Eastern Cooperative Oncology Group, *RT:* radiotherapy, *GTV:* gross tumor volume, *CIRT: *carbon ion radiotherapy

* American Joint Committee on Cancer Staging, 8th Edition; # One patient with rare pathological type cannot be staged (endovascular sarcoma), and the size of tumor should be recorded: primary lesion is 4.7 cm and the largest metastatic lymph node is 2.1 cm

### CIRT and sequential systemic therapy

Among 33 patients, the methods used for breathing control were ABC in 2 patients, respiratory gating in 14 patients, and FB in 17 patients.

Ten patients received induction chemotherapy with or without immunotherapy before CIRT. The regimens included paclitaxel, docetaxel, carboplatin, cisplatin, doxorubicin, ifosfamide, cyclophosphamide, gemcitabine and sintilimab. Two patients received targeted therapy (anlotinib and imatinib) prior to CIRT. One patient received concurrent chemotherapy and targeted therapy during CIRT, with regimens of irinotecan, vincristine and anlotinib. One patient received immunotherapy (sintilimab) during CIRT. All systemic therapy was administered for disease control in locally advanced or aggressive tumors, rather than as adjuvant treatment. Thirty-three lesions received a total dose of 59.5–80.0 Gy (RBE) per 16–23 fractions, nine of these lesions received SIB of 64.6–82.8 Gy (RBE). Another two lesions received hypofractionated CIRT of 60–65 Gy (RBE) per 10 fractions. The median BED of all the 35 lesions was 100.8 (range 78–112.6) Gy (RBE). The treatment mode is detailed in Table 1.

### Toxicities

All patients tolerated CIRT well. No grade > 3 toxicity was observed. The only two grade 3 toxicities were acute chest pain and late brachial plexus injury. Grade 2 acute toxicities included oesophagitis (3 cases), pneumonitis (3), chest pain (1) and haematological toxicities (5). Grade 2 late toxicities included radiation-induced lung injury (RILI) (1 case), rib fracture (1), chest pain (1) and brachial plexus injury (1) (Table 2).

**Table 2:** Toxicities of the entire cohort

Acute toxicity	Grade (percentage, %)			
	1	2	3	4 ~ 5
Chest pain	0 (0.0)	1 (3.0)	1 (3.0)	0 (0.0)
Esophagitis	9 (27.3)	3 (9.1)	0 (0.0)	0 (0.0)
Pneumonitis	2 (6.1)	3 (9.1)	0 (0.0)	0 (0.0)
Leukocytopenia	2 (6.1)	3 (9.1)	0 (0.0)	0 (0.0)
Neutropenia	5 (15.2)	2 (6.1)	0 (0.0)	0 (0.0)
Dermatitis	8 (24.2)	0 (0.0)	0 (0.0)	0 (0.0)
Pleural effusion	3 (9.1)	0 (0.0)	0 (0.0)	0 (0.0)
Cough	2 (6.1)	0 (0.0)	0 (0.0)	0 (0.0)
Thrombocytopenia	2 (6.1)	0 (0.0)	0 (0.0)	0 (0.0)

Late toxicity	Grade (percentage, %)			
	1	2	3	4 ~ 5
Brachial plexus injury	0 (0.0)	1 (3.0)	1 (3.0)	0 (0.0)
RILI	11 (33.3)	1 (3.0)	0 (0.0)	0 (0.0)
Rib fracture	0 (0.0)	1 (3.0)	0 (0.0)	0 (0.0)
Chest pain	0 (0.0)	1 (3.0)	0 (0.0)	0 (0.0)
Pleural effusion	3 (9.1)	0 (0.0)	0 (0.0)	0 (0.0)
Upper limb edema	2 (6.1)	0 (0.0)	0 (0.0)	0 (0.0)
Skin	2 (6.1)	0 (0.0)	0 (0.0)	0 (0.0)

RILI: radiation-induced lung injury

### Clinical outcomes

By the last follow-up on January 31st, 2025, the median follow-up time was 42.1 (range: 3.8–100.6) months for all patients and 40.7 (range: 3.8–100.6) months for patients with primary thoracic BSTS. The overall response rate (ORR) in all 35 lesions after CIRT was 65.7%, with complete response (CR) in 11, partial response (PR) in 12, and stable disease (SD) in 12 lesions. The 3-year LC rate was 85.2%. There was no statistically significant difference in LC when categorized by median gross tumor volume (≥ 111.41 cm^3^ vs. < 111.41 cm^3^, 87.5% vs. 84.6%, *p* = 0.855) and median BED (≥ 100.8 Gy (RBE) vs. < 100.8 Gy (RBE), 93.8% vs. 77.9%, *p* = 0.341). Two of six lesions with previous thoracic RT history experienced recurrence after a CIRT dose of 60–70 Gy (RBE), whereas only 3 recurrences occurred among 29 lesions without previous thoracic RT history. Notably, none of the 9 lesions treated with SIB technique experienced recurrence, while 5 recurrences were observed among 26 lesions not receiving SIB.

**Fig. 2:**
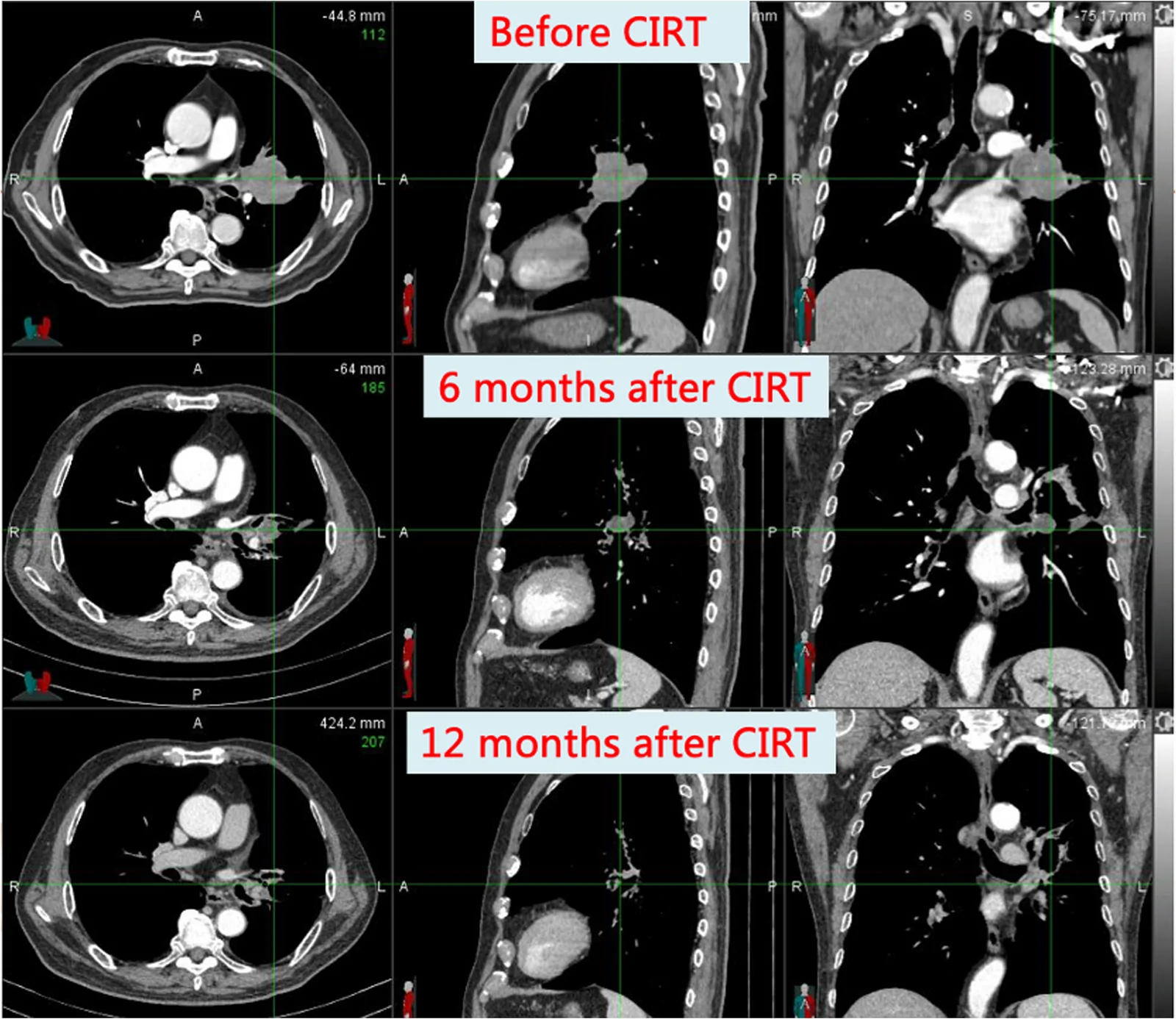
A typical case. A typical case of synovial sarcoma located in the left lung. The patient was treated with carbon ion radiotherapy of 69 Gy (RBE) in 23 fractions (with a simultaneous integrated boost to 75.9 Gy (RBE) in 23 fractions). The tumour shrank significantly 12 months after carbon ion radiotherapy and was evaluated as partial response

**Fig. 3:**
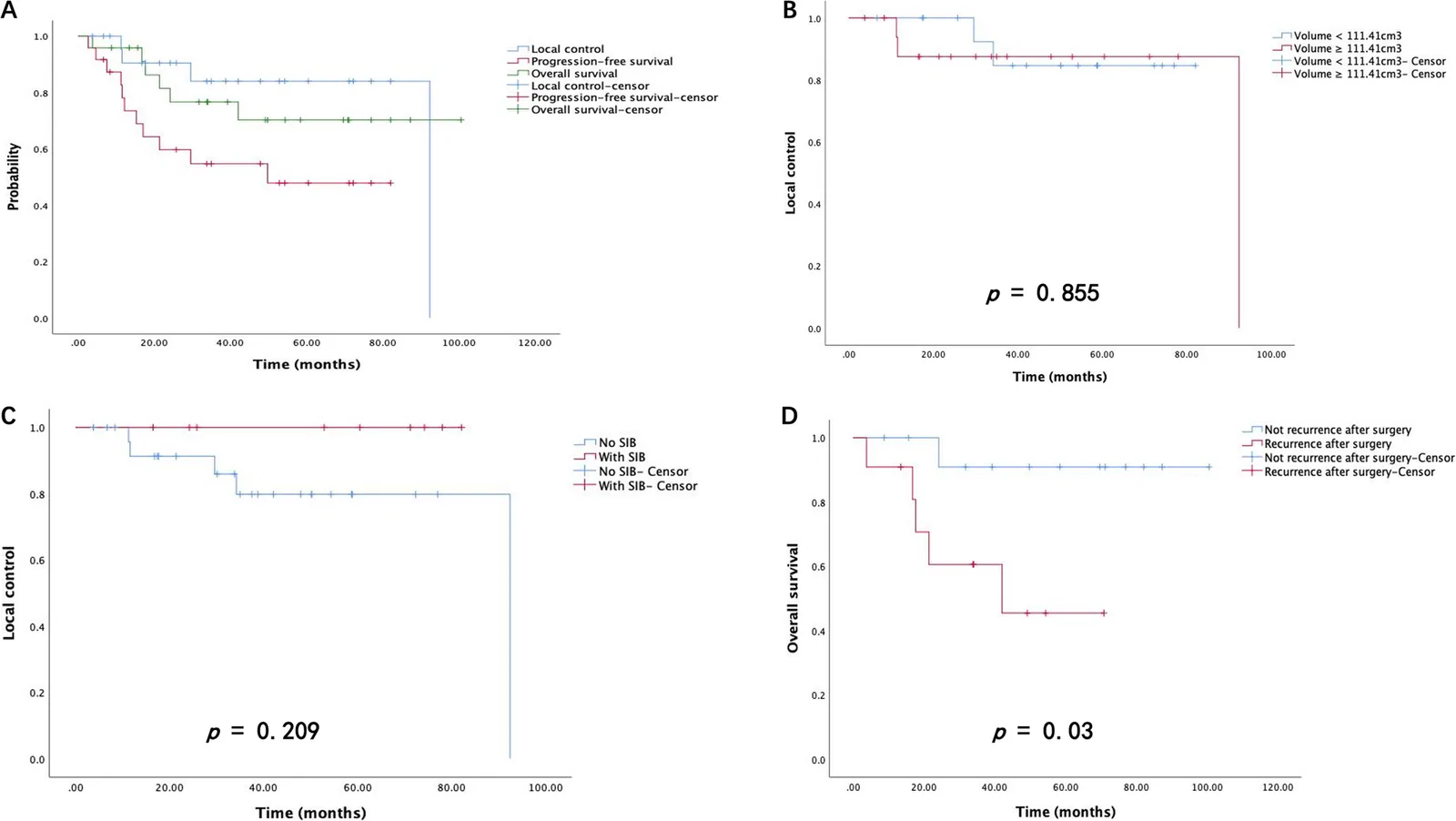
Survival outcomes of patients with thoracic bone and soft tissue sarcomas (BSTS). **A**: Local control (LC), progression-free survival (PFS), and overall survival (OS) in 24 patients with primary BSTS treated with carbon ion radiotherapy. **B**: LC stratified by tumor volume for the entire cohort (*n* = 35). **C**: LC stratified by the use of simultaneous integrated boost (SIB) for the entire cohort (*n* = 35). **D**: OS stratified by the presence of postoperative recurrence (PORC) in 24 patients with primary BSTS

In the subgroup of 24 patients with primary BSTS (24 lesions), the 3-year LC, PFS, and OS rates were 84%, 54.8%, and 76.7%, respectively. The results of the LC analysis were similar to those of the entire cohort. When stratified by BED and tumor volume, no intergroup differences were observed. Two of three patients with thoracic RT history experienced recurrence, whereas only 2 recurrences occurred among 21 patients without thoracic RT history. None of the 6 patients treated with SIB technique experienced recurrence, while 4 recurrences were observed among 18 patients not receiving SIB. Patients received systemic therapy had worse LC (no systemic therapy vs. received systemic therapy, 100% vs. 58.3%, *p* = 0.023) and PFS (no systemic therapy vs. received systemic therapy, 69.2% vs. 30.0%, *p* = 0.044). Additionally, PORC was associated with worse PFS (no PORC vs. PORC, 72.7% vs. 36.4%, *p* = 0.044) and OS (no PORC vs. PORC, 90.9% vs. 75.0%, *p* = 0.03) (Table 3). A typical case is shown in Fig. 2. The 3-year LC, PFS, and OS of patients with primary thoracic BSTS and stratified by PORC are shown in Fig. 3.

**Table 3:** Predicting local control, progression-free survival, and overall survival using different parameters in entire cohort and subgroup of primary thoracic bone and soft tissue sarcoma

Parameters	Local control			Progression-free survival			Overall survival		
Entire cohort (33 patients, 35 lesions)#									
BED of CIRT (median = 100.8 Gy (RBE))	≥Med	< Med	p*	N/A			N/A		
	93.8%	77.9%	0.341						
GTV volume (median = 111.41 cm^3^)	≥Med	< Med	p*						
	87.5%	84.6%	0.855						
Dose of CIRT (median = 70.4 Gy (RBE))	≥Med	< Med	p*						
	87.4%	83.1%	0.665						
Use SIB or not	SIB	No SIB	p*						
	100%	79.8%	0.209						
	0 / 9	5 / 26							
Thoracic radiotherapy history or not	Yes	No	p*						
	83.3%	85.5%	0.711						
	2 / 6	3 / 29							
Subgroup of primary thoracic bone and soft tissue sarcoma (24 patients, 24 lesions)									
BED of CIRT (median = 100.8 Gy (RBE))	≥Med	< Med	p*	≥Med	< Med	p*	≥Med	< Med	p*
	90.0%	79.5%	0.639	64.6%	45.5%	0.242	71.8%	68.2%	0.958
GTV volume (median = 130.0 cm^3^)	≥Med	< Med	p*	≥Med	< Med	p*	≥Med	< Med	p*
	80.0%	87.5%	0.454	55.0%	53.5%	0.805	71.3%	68.2%	0.931
Dose of CIRT (median = 70.4 Gy (RBE))	≥Med	< Med	p*	≥Med	< Med	p*	≥Med	< Med	p*
	90.0%	79.5%	0.639	45.5%	55.0%	0.242	71.8%	68.2%	0.958
Use SIB or not	SIB	No SIB	p*	SIB	No SIB	p*	SIB	No SIB	p*
	100%	78.0%	0.268	100%	78%	0.268	83.3%	64.9%	0.435
	0 / 6	4 / 18							
Thoracic radiotherapy history	Yes	No	p*	Yes	No	p*	Yes	No	p*
	66.7%	86.6%	0.288	66.7%	52.9%	0.644	100%	65.5%	0.29
	2 / 3	2 / 21							
PORC	Yes	No	p*	Yes	No	p*	Yes	No	p*
	80.0%	90.0%	0.347	36.4%	72.7%	**0.049**	45.5%	90.9%	**0.03**
System therapy	Yes	No	p*	Yes	No	p*	Yes	No	p*
	58.3%	100%	**0.023**	30.0%	69.2%	**0.044**	75.0%	68.4%	0.780

*Abbreviations*: *BED:* biological effective dose, *CIRT:* carbon-ion radiotherapy, *N/A:* not applicable, *Med:* median, *GTV:* gross tumor volume, *SIB:* simultaneous integrated boost, *PORC:* postoperative recurrence

*p value of Log-rank test; # Analyses of progression-free survival and overall survival were not applicable for the entire cohort

Values in bold indicate statistically significant differences (*p* < 0.05)

## Discussion

### Summary of key findings

To our best knowledge, this is the first study to report the treatment toxicities and outcomes of CIRT for primary or metastatic thoracic BSTS. Notably, this modality yielded a 3-year LC of 85.2% with a favorable safety profile (only 6.1% Grade 3 toxicity and no ≥ Grade 4 events) at doses up to 80 Gy (RBE). Furthermore, this study identified several potential key predicting factors: a history of prior thoracic RT predicted poorer LC, while the use of a SIB technique was associated with improved LC; In patients with primary thoracic BSTS, systemic therapy correlated with inferior LC and PFS, and postoperative recurrence (PORC) portended worse PFS and OS. Despite the influence of various predicting factors, the manageable toxicity profile coupled with encouraging LC rates in this cohort supports PBS-CIRT as a viable therapeutic option for unresectable thoracic BSTS.

### Photon radiotherapy for BSTS

Photon radiotherapy demonstrates limited efficacy in BSTS. In a study by Maldegem et al. [10] involving 171 patients with advanced, unresectable conventional central chondrosarcoma, tumors were located in the extremities (41%), pelvis (37%), ribs/scapulae (12%), vertebral bodies (5%), and other sites (5%). Delivered doses ranged from 24 Gy/3 fractions to 66 Gy/33 fractions. Survival rates declined sharply from 48% at 1 year to only 2% at 5 years, with a median OS of 11 months. LC outcomes were also poor, with an earlier study reporting a 2-year local control rate of merely 39% [11].

In contrast, Kepka et al. [12] treated 112 soft tissue sarcoma patients with higher radiation doses (median: 64 Gy, range: 25–87.5 Gy). Tumor sites included extremities (43%), retroperitoneum (26%), head and neck (24%), and trunk wall (7%). A 5-year LC rate of 60% was achieved in the subgroup receiving ≥ 63 Gy. However, with dose escalation, the incidence of severe (grade ≥ 3) radiotherapy-related toxicities—defined as life-threatening, organ failure, or requiring major surgical intervention (e.g., grafting or amputation)—rose significantly: 27% in the ≥ 68 Gy group versus 8% in the < 68 Gy group. Modern RT techniques including helical tomotherapy, volumetric modulated arc therapy and stereotactic radiotherapy (SBRT) still yield LC rates below 60%, and the incidence of toxicities of grade 3 or higher reaches 26% [2]. SBRT has demonstrated excellent LC in metastatic BSTS, with reported 2-year LC as high as 98.3%. This favorable outcome may be partly explained by the relatively small tumor burden in the studied cohort (median maximum tumor diameter was 3.4 cm). However, the clinical application of SBRT remains limited in many scenarios due to concerns over severe radiotherapy-related toxicities [13].

### Efficacy and safety of CIRT

CIRT has demonstrated promising efficacy in BSTS, with reported 5-year LC and OS rates ranging from 65% to 100% and 46% to 53%, respectively [4, 14–16]. In a study of 128 patients with unresectable axial sarcomas, Imai R et al. [4]. reported 5-year LC, OS, and disease-free survival rates of 65%, 46%, and 39%, respectively (median follow-up: 49.4 months; median tumor volume: 356 cm³). Similarly, Matsumoto K et al. [15] observed 5-year LC, OS, and PFS rates of 79%, 52%, and 48% among 47 patients with vertebral sarcomas (median tumor volume: 190 cm³; median follow-up: 25 months). In that study, lesions located < 5 mm from the spinal cord or with volume > 100 cm³ were associated with increased recurrence risk. Our results in thoracic BSTS are consistent with these findings. The 3-year LC rate for the entire cohort was 85.2%, while among patients with primary tumors, 3-year LC and OS reached 84.0% and 76.7%, respectively. These outcomes help address the existing gap in evidence regarding CIRT for thoracic BSTS.

CIRT yields favorable therapeutic outcomes with mild and acceptable radiotherapy-related adverse reactions. In a study by Matsumoto et al. [15], 1 case of acute grade 3 radiation-induced skin reaction was reported; 2 patients developed grade 3–4 late-stage skin reactions. Seven cases of vertebral compression fractures were documented, and the incidence of compression fractures was significantly higher in patients who received a radiation dose of 70.4 Gy (RBE) than in those who received a dose lower than 70.4 Gy (RBE) (*p* = 0.0331). A study by Imai R et al. [4] reported that 4 patients experienced late adverse events of grade ≥ 3, including spinal cord injury, peripheral nerve injury, colon injury, and skin injury, with an incidence rate of 2.3%. Our study also indicated a low incidence of adverse reactions, with only 2 cases of grade ≥ 3 adverse reactions observed (6.1%).

Although Japanese researchers have recently proposed dose constraints for normal tissues in CIRT [17], their widespread clinical adoption remains challenging. Significant variations in RBE, complexities in calculating the BED, and differences in both technical facilities and underlying biological models (e.g., the Microdosimetric Kinetic Model (MKM) used in Japan versus the LEM-I employed at our institution) hinder the establishment of universal dose constraints [18, 19]. Consequently, protocols and constraints are not directly interchangeable between centers, underscoring the need for institution-specific guidelines to optimally protect organs at risk and minimize severe toxicity. In our study, under the current institution-specific dose constraint framework, the incidence of grade ≥ 3 adverse events was 6.1% in this cohort. These findings provide preliminary, model-specific guidance for centers using the LEM-I and may inform the future development of standardized consensus protocols.

### Dose, tumor volume, and SIB technique

Improving LC is critical in the treatment of BSTS due to its direct association with OS [20, 21]. However, the factors influencing LC in CIRT are multi‑faceted and not fully established. A notable debate exists regarding the impact of tumor volume on LC. Although Jingu et al. [22] found no significant difference in LC using a 100 cm³ cutoff, other studies have reported significantly reduced LC with volumes exceeding 300 cm³ [23], 460 cm³ [4], and 1000 cm³ [24]. In our cohort, no significant difference in LC was observed using thresholds of 111.4 cm³ (entire cohort) or 130.0 cm³ (primary thoracic BSTS subgroup), aligning with the findings of Jingu et al.

A dose-response relationship for LC has been demonstrated in CIRT. For instance, in liposarcoma patients treated with protons and carbon ions, the 3-year LC was significantly higher in those receiving doses ≥ 72.7 Gy (EQD₂) compared to those receiving lower doses (79.1% vs. 20%) [25]. In our study, a trend toward better LC was observed in the group with a BED ≥ 100.8 Gy (RBE) (93.8% vs. 77.9%), although this difference did not reach statistical significance (*p* = 0.341), likely due to the limited sample size.

BED was calculated using the linear‑quadratic model (α/β = 10 Gy) to standardize comparisons across different fractionation schedules. Given the absence of clinically validated BED cutoff values for CIRT in thoracic BSTS, we dichotomized BED at the median for exploratory purposes only. This median value was chosen for statistical convenience to balance subgroup sizes and does not imply a clinically optimal threshold. We therefore emphasize that this threshold is hypothesis‑generating, and future studies are needed to establish validated, clinically meaningful BED cutoffs for CIRT in this disease setting.

A key finding was the potential benefit associated with dose escalation using the SIB technique. Remarkably, none of the 9 lesions treated with SIB experienced recurrence during the follow-up period, whereas all 5 recurrences occurred in the 26 lesions treated without SIB. Although this numerical difference did not reach statistical significance (*p* = 0.209), likely due to the limited sample size, it suggests a clinically meaningful trend. The SIB technique selectively escalates the dose to the tumor core and increases the mean dose to the target volume, resulting in a significantly higher median BED in the SIB group (104.9 Gy (RBE) vs. 94.5 Gy (RBE), *p* = 0.0086). In addition, SIB may potentially enhance dose-averaged linear energy transfer (LETd) distribution within the tumor [26], without increasing the dose to surrounding OARs, which differs from photon RT, where dose escalation is frequently accompanied by elevated toxicity risks. Notably, both grade 3 toxicities occurred in the non-SIB group, indicating that dose intensification via SIB did not translate into increased severe adverse events. Taken together, the favorable LC trend observed in the SIB group is more likely attributable to the higher radiation dose delivered rather than the SIB technique per se, with optimized LETd distribution potentially serving as an additional contributing factor [27].

### Other prognostic factors

Patients with a history of thoracic RT showed inferior LC, consistent with findings from our institutional studies on head and neck sarcomas [28]. This may be related to the lower doses delivered in re‑irradiation settings (median BED: 92.1 vs. 100.9 Gy (RBE) in non‑RT history group, *p* = 0.0029). Based on our data, a BED ≥ 100.8 Gy (RBE) is recommended to achieve satisfactory LC in thoracic BSTS treated with CIRT. In our cohort, patients with PORC had significantly worse PFS and OS, aligning with prior reports that link PORC to higher disease‑specific mortality [21, 29, 30]. While some earlier studies did not confirm this association [31, 32], recent evidence emphasizes that recurrence after unplanned excision is an adverse prognostic factor, underscoring the importance of appropriate surgical planning [33].

The heterogeneity of systemic therapy in this cohort (encompassing diverse drug categories, regimens, timing, and treatment intent) arises primarily from the rarity and histopathological diversity of thoracic BSTS, as well as the retrospective nature of study design. The inferior LC and PFS observed in patients receiving systemic therapy largely reflected selection bias, as such treatment was preferentially administered to those with larger tumors, higher histological grade, more aggressive biology, or recurrent disease—all characteristics inherently associated with poorer prognosis [34, 35]. This heterogeneity may further confound survival analyses and limit causal inference. Well‑designed prospective studies with standardized systemic therapy protocols are needed to definitively clarify its role when combined with CIRT for thoracic BSTS.

### Histological heterogeneity and the desmoid subgroup

Our cohort included various histological subtypes of BSTS with potentially distinct radiosensitivity. Radiosensitive subtypes, such as myxoid liposarcoma, typically achieve satisfactory LC with conventional photon RT. However, the unique physical and biological properties of CIRT may offer particular advantages for less radiosensitive subtypes. Unlike photons, carbon ions deposit energy with a high linear energy transfer (LET), leading to increased complex DNA double-strand breaks that are less repairable by tumor cells. This high LET enables CIRT to overcome intrinsic radioresistance more effectively than conventional photon therapy. Haas et al. [36]. reported that the surviving fraction at 2 Gy (SF2) for 14 sarcoma cell lines ranged from 0.27 (synovial sarcoma) to 0.76 (leiomyosarcoma), with a mean of 0.52. The median α/β ratio was 4.9 Gy, and 6 cell lines had an α/β ratio below 4 Gy, suggesting that hypofractionated radiotherapy (dose per fraction > 2 Gy) may be advantageous for BSTS. Consequently, grouping of diverse histologic subtypes may obscure subtype‑specific differences in treatment response and limit the precision of our outcome estimates. In the present study, the single‑fraction dose of CIRT (3–6.5 Gy [RBE]) was substantially higher than conventional fractionation. The combination of high LET, enhanced RBE, and hypofractionated delivery may explain the favorable LC achieved even for relatively radioresistant subtypes.

Nevertheless, the inclusion of diverse histologic subtypes in our cohort presents an important caveat. Grouping these subtypes may obscure subtype‑specific differences in treatment response and limit the precision of our outcome estimates. Therefore, our reported LC rates should be interpreted as an average effect across a heterogeneous cohort, and individualized treatment decisions should consider histology‑specific radiosensitivity. Future studies using xenograft models, genomic analyses, and large‑scale clinical trials are needed to better stratify patients and identify those most likely to benefit from CIRT.

Further, aggressive fibromatosis is an intermediate (locally aggressive) soft tissue tumor, not a true malignant sarcoma, and shows favorable radiosensitivity. Photon RT at 56 Gy achieves a 3‑year local control rate of 81.5%, but young age (< 30 years) and large tumor volume increase local failure risk [37, 38]. Following exclusion of four patients with aggressive fibromatosis (all primary thoracic cases), the 3‑year LC for all lesions was 83.5%. For primary thoracic BSTS, the 3‑year LC, PFS, and OS were 80.8%, 47.2%, and 65.4%, respectively. The higher OS in the full cohort (76.7%) confirmed the more favorable prognosis of desmoid tumors.

### Limitations and future directions

This study has several limitations. First, the retrospective design and limited cohort size (33 patients, comprising 35 lesions for LC analysis and 24 primary thoracic BSTS cases for LC, PFS, and OS assessments) precluded multivariate analysis and may substantially limit the generalizability of our findings. Second, the 26 uncorrected univariate log‑rank tests (5 overall, 21 subgroup) yielded an approximate 74% probability of at least one false‑positive at α = 0.05. Subgroup findings are exploratory and require large‑scale validation; p‑values are tentative due to type I error inflation. Third, the inclusion of both primary and metastatic sarcomas introduces potential confounding, as these tumor types differ in biology, prognosis, and therapeutic intent. Fourth, our study included a variety of histologic subtypes with potentially heterogeneous radiosensitivity. Fifth, the inclusion of patients with PORC or prior thoracic RT, populations with likely distinct tumor biology and radiosensitivity, may have further contributed to confounding. In addition, tumor locations were heterogeneous in the present study. Given the rarity of thoracic BSTS, prospective randomized trials remain challenging. Nevertheless, with the increasing availability of carbon-ion therapy centers, future multi‑institutional collaborations supporting larger prospective studies with homogeneous histology or site-specific designs are warranted to draw more definitive conclusions regarding CIRT for thoracic BSTS.

## Conclusions

This first retrospective analysis of CIRT for thoracic BSTS reports an unadjusted 3‑year local control rate of 85.2% with mild CIRT-related toxicities in a small single‑institution cohort, suggesting potential efficacy. Toxicity was manageable under LEM‑based constraints, offering preliminary guidance for other centers. However, limitations include small sample size and uncorrected multiple comparisons (inflated type I error), making results exploratory and inconclusive. Larger multi‑institutional prospective studies with standardized histology and site‑specific stratification are needed to validate these findings.

## Data Availability

The datasets used in the current study available from the corresponding author on reasonable request.

## Abbreviations

4-D CT −: 4-Dimensional Computed Tomography
ABC: Active Breathing Control
BED: Biological Effective Dose
BSTS: Bone and Soft Tissue Sarcoma
CR: Complete Response
CIRT: Carbon Ion Radiotherapy
CT: Computer Tomography
CTCAE: Common Terminology Criteria for Adverse Events
CTV: Clinical Target Volume
ECOG: Eastern Cooperative Oncology Group
EQD₂: Equivalent Dose in 2 Gy Fractions
FB: Free Breathing
FDG: PET/CT-Fluorodeoxyglucose Positron Emission Tomography/Computed Tomography
FNCLCC: Fédération Nationale des Centres de Lutte Contre le Cancer
GTV: Gross Tumor Volume
Gy (RBE): Gray of relative biological effectiveness weighted dose
iGTV: Internal Gross Tumor Volume
IRB: Institutional Review Board
LC: Local Control
LEM: Local Effect Model
LETd: Dose-Averaged Linear Energy Transfer
L-Q model: Linear-Quadratic model
MKM: Microdosimetric Kinetic Model
MRI: Magnetic Resonance Imaging
OARs: Organs at Risk
ORR: Overall Response Rate
OS: Overall Survival
PBT: Proximity Bronchial Tree
PFS: Progression-Free Survival
PORC: Postoperative Recurrence
PR: Partial Response
PTV: Planning Target Volume
RBE: Relative Biological Effectiveness
RECIST: Response Evaluation Criteria in Solid Tumors
RILI: Radiation-Induced Lung Injury
RM: Residual Motion
RT: Radiotherapy
SD: Stable Disease
SIB: Simultaneous Integrated Boost
SPHIC: Shanghai Proton and Heavy Ion Center
XRT: Photon Radiotherapy
